# Skipping breakfast leads to weight loss but also elevated cholesterol compared with consuming daily breakfasts of oat porridge or frosted cornflakes in overweight individuals: a randomised controlled trial

**DOI:** 10.1017/jns.2014.51

**Published:** 2014-11-13

**Authors:** Allan Geliebter, Nerys M. Astbury, Roni Aviram-Friedman, Eric Yahav, Sami Hashim

**Affiliations:** 1 New York Obesity Nutrition Research Center, St Luke's-Roosevelt Hospital Center, New York, NY, USA; 2 Institute of Human Nutrition, Columbia University College of Physicians and Surgeons, New York, NY, USA; 3 Department of Psychiatry, Columbia University Medical Center, New York, NY, USA

**Keywords:** Breakfast, Body weight, Oats, Cholesterol, REE, resting energy expenditure

## Abstract

Eating breakfast may reduce appetite, body weight and CVD risk factors, but the breakfast type that produces the greatest health benefits remains unclear. We compared the effects of consuming a high-fibre breakfast, a non-fibre breakfast, or no-breakfast control on body weight, CVD risk factors and appetite. A total of thirty-six overweight participants (eighteen men and eighteen women) (mean age 33·9 (sd 7·5) years, mean BMI 32·8 (sd 4·7) kg/m^2^) were randomly assigned to consume oat porridge (*n* = 12), frosted cornflakes (*n* = 12) or a water control (*n* = 12) breakfast daily for 4 weeks. Appetite ratings were collected on the first day and weekly thereafter. Before and after the intervention, body weight, composition, blood pressure and resting energy expenditure (REE) were measured and a fasting blood sample was collected. Across the 4 weeks, fullness was higher and hunger was lower in the oat porridge group compared with the control group (*P* < 0·05). Mean weight change over the intervention was significantly different in the control group (−1·18 (sd 1·16) kg) compared with both the cornflakes (−0·12 (sd 1·34) kg) and oat porridge (+0·26 (sd 0·91) kg) groups (*P* < 0·05). However, the control group also showed elevated total cholesterol concentrations relative to the cornflakes and oat porridge groups (*P* < 0·05). There were no differences between groups in changes in body composition, blood pressure, REE or other CVD risk factors. In conclusion, although skipping breakfast led to weight loss, it also resulted in increased total cholesterol concentrations compared with eating either oat porridge or frosted cornflakes for breakfast.

The proportion of individuals who regularly consume breakfast has declined in recent decades^(^^1^^)^, during which time there has been an increase in obesity prevalence^(^^2^^)^. Many epidemiological studies report that regular breakfast eaters tend to have a lower BMI than those who do not consume breakfast regularly^(^^3^^–^^7^^)^. Furthermore, regular breakfast consumption is associated with lower risk of CVD^(^^8^^)^, since consuming breakfast has been associated with a lower body weight, a reduction in total and LDL-cholesterol concentrations, lower blood pressure, and greater insulin sensitivity^(^^9^^,^^10^^)^, which are modifiable risk factors for CVD. However, despite many epidemiological studies reporting associations between the regular consumption of breakfast and various health outcomes, there are a limited number of randomised controlled trials that would establish a plausible cause–effect relationship^(^^11^^–^^13^^)^. Furthermore, some of the limited randomised controlled trials may be subject to biased research reporting^(^^14^^)^.

Some researchers suggest that since breakfast consumption increases fullness and suppresses hunger and desire to eat^(^^3^^,^^15^^)^, eating breakfast may decrease the likelihood of overeating or snacking later in the day^(^^11^^,^^16^^)^. What is more, if the reduction in subsequent energy intake is greater than the net energy of the breakfast, overall daily energy intake would be lower when breakfast was eaten than when it was omitted, which could result in weight loss. Alternatively, one could argue that skipping breakfast does not lead to a compensatory increase in subsequent energy intake, which may lead to lower overall daily energy intake and weight loss^(^^13^^)^.

The influence of differences in the composition of breakfast on changes in body weight has also not been adequately investigated using randomised controlled trials. Data from the Third National Health and Nutrition Examination Survey (NHANES III) show that individuals who eat a ready-to-eat cereal, cooked cereal, or quick-breads (muffins, pancakes, etc.) for breakfast have a significantly lower BMI than breakfast skippers or those who consume meat and eggs for breakfast^(^^3^^)^. Some studies have reported that breakfasts high in fibre reduce subjective appetite more than those low in fibre^(^^17^^,^^18^^)^. Oats are high in dietary fibre, including the soluble fibre β-glucan^(^^19^^)^, which has been shown to reduce hypercholesterolaemia^(^^20^^)^.

The aim of the present study was to investigate the effects of consuming a high-fibre oat porridge, an isoenergetic non-fibre frosted cornflakes breakfast, and a no-breakfast water control daily for 4 weeks on body-weight changes, subjective appetite and CVD risk factors in overweight but otherwise healthy individuals.

## Participants and methods

### Study design

We conducted a randomised, controlled parallel-arm study at the New York Obesity Nutrition Research Center at St Luke's Hospital. Volunteers were screened, and eligible candidates were enrolled and randomly assigned to one of three breakfast groups: (1) oat porridge (*n* = 12; six men and six women); (2) frosted cornflakes (*n* = 12; six men and six women); or (3) a no-breakfast control group (*n* = 12; six men and six women). Participants, stratified by sex, were randomised to the experimental groups using equal-sized blocks, with subsequent replacement as needed for dropouts. The present study was conducted according to the guidelines laid down in the Declaration of Helsinki, and all procedures involving human subjects were approved by St Luke's Roosevelt Hospital Center Institutional Review Board. Written informed consent was obtained from all participants, who received a financial incentive for taking part in the study. Data were collected during 1998 and 1999. The present study was registered at clinicaltrials.gov as NCT02035150.

### Participants

Overweight (BMI > 25 kg/m^2^) candidates were included in the study if they were aged 18–65 years, weight stable (<5 % change in body weight during the past 3 months), and free from chronic disease. They were excluded if they smoked, took medications regularly, currently or previously abused drugs or alcohol, or were currently or intending to undertake a weight-loss diet or exercise programme. Females were excluded if they were pregnant or lactating. Participants were asked not to make changes to their physical activity pattern during the study.

A total of forty-six eligible participants were recruited from the general population of New York City via newspaper advertisements and flyers and enrolled in the study. A total of five participants dropped out during the course of the study (two men and three women), and replacements were recruited. Thus thirty-six participants (eighteen men and eighteen women) completed the study and were included in the final analyses. Participant demographics are displayed in Table 1.

**Table 1.** Physical characteristics of study participants (Mean values and standard deviations)

	Males (*n* 18)		Females (*n* 18)		All (*n* 36)	
	Mean	sd	Mean	sd	Mean	sd
Age (years)	35·6	6·1	32·3	8·6	33·9	7·5
Body weight (kg)	96·1	11·7	91·0	16·6	93·5	14·5
BMI (kg/m^2^)	32·2	3·4	34·5	5·4	32·9	4·7
Body fat (kg)*	32·7	10·6	40·5	11·4	36·6	11·6
Lean mass (kg)†	63·4	6·7	50·6	6·5	57·0	9·2

*Measured by air displacement (BOD POD^®^).

†Calculated as the difference between total body weight and fat mass measured using air displacement.

### Protocol

During the study, participants were required to report to the hospital at 08·30 hours following an overnight fast (>10 h) on each weekday for 4 consecutive weeks. On arrival, they were given 15 min to consume their assigned breakfast, which either consisted of oat porridge, frosted cornflakes or no-breakfast (control) (Table 2). The oat porridge breakfast consisted of oat porridge (Quick Oats; Quaker) made with whole milk (3·25 % fat) and heated in a microwave oven (according to the manufacturer's instructions) immediately before serving. The cornflakes breakfast consisted of frosted cornflakes (Frosted Flakes; Kellogg's) served with low-fat (1·7 % fat) milk, and the control breakfast consisted of 350 ml water in a cup. The volumes of the oat porridge and cornflakes breakfast meals were matched to the control breakfast by adding 230 ml water into the oat porridge breakfast, and by serving 190 ml water on the side in a cup for the cornflakes breakfast. All participants also received 200 ml of decaffeinated coffee with 12 ml non-dairy creamer (Coffee Mate; Nestlé) and a 1 g packet of artificial sweetener (Equal; The NutraSweet Company) to consume with their allocated breakfast. On Fridays, participants were provided with two portions of the breakfasts to take home and consume on the weekend days. They were asked to return the empty containers at the beginning of the following week to ensure compliance. On the first day of the intervention and at weekly intervals thereafter, subjective appetite ratings in response to the breakfasts were collected for 3 h. The laboratory assessments were undertaken before the start, and a day after the end of the 4-week intervention.

**Table 2.** Nutritional composition of the breakfasts provided during the intervention

	Control	Cornflakes	Oat porridge
Energy			
kJ	44	1473	1472
kcal	11	352	351
Total (non-fibre) carbohydrate			
g	1·0	66·3	60·6
% Energy	38	75	69
Which included			
Sugars (g)	0·8	35·5	8·9
Protein			
g	0	7·3	14·3
% Energy	0	8	16
Fat			
g	0·5	5·7	6·6
% Energy	43	15	17
Fibre (g)	0	0	8·0*

*4 g soluble fibre and 4 g insoluble fibre.

### Procedures, laboratory visits and assessments

Participants were asked to fast overnight (>10 h) and report to the laboratory at 08·30 hours. The laboratory technicians undertaking the pre and post study assessments were blinded to the experimental group of the participants. Pre and post anthropometrics, body composition, blood pressure and resting energy expenditure (REE) were assessed before a fasting venous blood sample was collected. The research assistant was not blinded to the appetite ratings collected during the study.

#### Anthropometric measurements

Participants were asked to void and to remove all outer clothing and shoes before weight was measured to the nearest 0·1 kg using an electronic scale (Weight-Tronix Inc.). Height was measured to the nearest 1 cm using a stadiometer (Holtain). Participants stood with their feet 25–30 cm apart, and their waists were measured with a flexible nylon tape to the nearest 0·1 cm in a horizontal plane midway between the lower margin of the last rib and the iliac crest^(^^21^^)^. Body composition was estimated using air displacement plethysmography (BOD POD®; Life Measurements Inc.). Participants changed into a swimsuit and a swim cap before being seated inside the BOD POD. Total body volume was estimated twice, and the average was used to determine body fat mass based on the manufacturer built-in equations. Fat-free mass was calculated as the difference between total body weight and body fat mass measured using air displacement.

#### Resting energy expenditure

REE measurements were collected using a metabolic cart (Sensormedics). Standard gas calibrations were performed before each subject was tested. Participants rested comfortably on a bed in a semi-supine position, under thermoneutral conditions for 30 min, before a plastic transparent ventilated hood was placed over their head for 30 min. The first and final 5-min recordings were ignored, and the gas exchange results were converted to REE (kJ/d) using the formula by Weir^(^^22^^)^. Gas concentration measurements were reproducible to within 2·6 % for a standard alcohol phantom.

#### Blood pressure

Blood pressure readings were obtained by sphygmomanometer. Participants rested for 15 min before blood pressure measurements were obtained. Systolic and diastolic measurements were recorded as phase I and V Korotkoff sounds^(^^23^^)^. Three separate blood pressure measurements were obtained at 2-min intervals, and the means of the three values for both systolic and diastolic blood pressure were used for analysis.

#### Blood sample collection and analysis

A fasting blood sample was collected from an antecubital vein in the less dominant forearm using venepuncture. Samples were drawn into serum-separating tubes and allowed to clot for 30 min at room temperature before being centrifuged at 3000 ***g*** for 15 min at 4°C. Samples were stored at 4°C until analysis. Serum glucose, protein-bound glucose, insulin and lipids (total cholesterol, TAG and HDL) were assayed at a Center for Disease Control-certified chemistry laboratory (Quest Diagnostics). LDL-cholesterol was calculated using the Friedewald equation, based on the values obtained from total cholesterol, HDL-cholesterol and TAG^(^^24^^)^. Homeostasis model assessment for insulin resistance (HOMA-IR) was calculated from fasting glucose and insulin^(^^25^^)^. Leptin was determined in-house by RIA (Linco Research), with an intra-assay CV of 5·3 % and an inter-assay CV of 7·3 %.

#### Subjective appetite ratings

Ratings of hunger and fullness were obtained using a Likert-type rating scale. Participants were asked to select the number which best reflected their current hunger and fullness based on a line with anchor points at: 0, not at all; 20, slightly; 40, moderately; 60, quite; 80, very; and 100, extremely. Appetite ratings were collected when participants arrived at the laboratory immediately before breakfast (baseline), immediately following the consumption of the breakfast (0 min) and at 30, 60 90, 120, 150 and 180 min later. Participants completed appetite ratings at these times on the first day of the intervention and weekly thereafter.

### Statistical analyses

SPSS software (version 21; IBM) was used for data entry and analysis. All data are reported as mean values with their standard errors unless otherwise stated. Sex was examined as a factor on the outcome variables, but there were no significant differences between the men and women for any of the outcome variables; hence the data were pooled.

Between-group comparisons of the pre-intervention data were performed using ANOVA with breakfast groups as a between-subjects factor. Post-intervention outcomes and the changes in the outcome variables from pre- to post-intervention were analysed using ANCOVA with the pre-intervention measure as a covariate. If significant effects were observed between the groups, *post hoc* pairwise comparisons were made by Fisher's least significant difference (LSD) test.

The pre-breakfast appetite ratings for each week were compared between the groups by ANOVA. There were significant differences in the pre-breakfast subjective hunger and fullness ratings between the groups; therefore the change from baseline was used in subsequent analyses. The changes from baseline subjective appetite ratings were examined using repeated-measures ANOVA, with week and time point as the within-subject factors and breakfast group as the between-subject factor. Significance was set at two-tailed *P* < 0·05.

## Results

### Physical characteristics

There were no between-group differences for any of the pre-intervention physical characteristics (Table 3). When controlling for initial body weight, there was a significant main effect of breakfast group for final body weight (*F*(2,32) = 6·4; *P* = 0·005) and change in body weight (*F*(2,32) = 6·0; *P* = 0·006). Pairwise comparisons revealed that final body weight was significantly lower in the control group than in both the cornflakes (*P* = 0·033) and oat porridge breakfast groups (*P* = 0·006), and change in body weight was greater in the control group compared with the cornflakes (*P* = 0·025) and oat porridge breakfast groups (*P* = 0·002). However, there were no significant differences between the cornflakes and oat porridge breakfast groups for final body weight or change in body weight over the intervention period when controlling for initial body weight. There were no significant between-group differences in final body fat mass, fat-free mass, waist circumference, waist:hip ratio, blood pressure and REE or the change in these variables over the intervention period when the pre-intervention measures were controlled (Table 3).

**Table 3.** Physical characteristics of the participants (Mean values and standard deviations)

Group… Sex… Ethnicity…	Control (*n* = 12) (6 male, 6 female) (4 W, 3 B, 3 H, 1 A, 1 O)						Cornflakes (*n* = 12) (6 male, 6 female) (6 W, 3 B, 2 H, 2 A, 1 O)						Oat porridge (*n* = 12) (6 male, 6 female) (6 W, 4 B, 1 H, 1 O)					
	Pre		Post		Change		Pre		Post		Change		Pre		Post		Change	
	Mean	sd	Mean	sd	Mean	sd	Mean	sd	Mean	sd	Mean	sd	Mean	sd	Mean	sd	Mean	sd
Body weight (kg)†	96·9	15·0	95·7	14·5	−1·2	1·2	88·8	14·0	88·7*	13·9	−0·1*	1·3	95·1	14·4	95·4*	14·1	0·3*	0·9
Waist (cm)	103·5	13·5	103·5	13·5	0·1	0·8	97·4	10·0	97·8	10·5	0·4	0·5	98·9	10·7	100·4	9·7	1·5	0·7
Waist:hip ratio	0·91	0·11	0·91	0·12	0·01	0·02	0·88	0·73	0·88	0·80	0·01	0·03	0·87	0·11	0·88	0·10	0·01	0·02
Fat mass (kg)‡	38·0	10·5	37·6	11·0	0·95	3·3	35·4	9·6	34·8	12·5	0·94	3·3	36·5	14·8	36·9	14·6	0·77	2·7
Fat-free mass (kg)§	58·9	8·4	58·0	6·9	−0·9	3·1	53·4	10·9	53·8	12·3	0·3	3·7	58·6	7·7	58·5	8·8	−0·1	2·7
Blood pressure:																		
Systolic (mmHg)	118·3	3·1	116·3	2·9	−2·0	7·9	117·0	3·1	117·8	2·9	0·8	4·0	119·5	3·1	119·3	2·9	−0·1	3·0
Diastolic (mmHg)	80·0	2·4	78·2	2·3	−1·7	5·5	73·3	2·4	73·6	2·3	0·2	6·0	75·5	2·4	78·3	2·3	2·8	8·2
REE (kJ/d)‖	8374	1577	7649	1507	−354	963	7322	1033	7180	1048	−24	552	8068	1901	8234	1867	−69	729

W, white; B, black; H, Hispanic; A, Asian; O, other; REE, resting energy expenditure.

*Mean value was significantly different from that of the control group (*P* < 0·05).

†ANCOVA using the pre-intervention measure as a covariate showed a significant effect of breakfast group for body weight (*P* = 0·006).

‡Measured by air displacement (BOD POD^®^).

§Calculated from the difference between total body weight and fat mass measured using air displacement.

‖REE assessed by indirect calorimetry using a ventilated hood and metabolic cart.

### Metabolic risk factors

There were no significant between-group differences in any of the pre-intervention metabolic risk factors (Table 4). There were no main effects of breakfast group for final values, or for the change from pre- to post-intervention in fasting serum glucose, protein-bound glucose, insulin, plasma leptin or HOMA-IR when pre-intervention values were controlled (Table 4).

**Table 4.** Fasting glucose, insulin and lipid profiles pre- and post-intervention and change over the intervention† (Mean values with their standard errors)

	Control						Cornflakes						Oat porridge					
	Pre		Post		Change		Pre		Post		Change		Pre		Post		Change	
	Mean	sem	Mean	sem	Mean	sem	Mean	sem	Mean	sem	Mean	sem	Mean	sem	Mean	sem	Mean	sem
Serum glucose (mmol/l)	5·1	0·1	5·0	0·1	−0·1	0·1	5·1	0·1	5·1	0·1	−0·04	0·2	4·8	0·1	5·0	0·1	0·2	0·1
Glycated albumin (mg/g)	0·97	0·02	0·9	0·02	−0·01	0·02	0·93	0·02	0·91	0·02	−0·02	0·01	0·96	0·02	0·93	0·02	−0·03	0·03
Serum insulin (pmol/l)	143·4	23·2	128·2	15·1	−15·1	15·0	134·3	16·4	132·0	15·2	−2·3	11·5	121·2	16·9	117·3	15·5	−7·1	6·5
Plasma leptin (μg/l)	21·5	4·6	19·5	4·2	−2·0	1·8	20·2	4·6	22·5	4·3	2·3	1·2	24·5	4·6	23·1	4·2	−1·4	2·3
HOMA-IR	4·7	0·8	4·1	0·5	−0·6	0·5	4·3	0·5	4·3	0·5	−0·1	0·3	3·8	0·6	3·8	0·6	0·1	0·2
Plasma cholesterol (mmol/l)																		
Total	5·6	0·5	5·9	0·5	0·4	0·1	5·3	0·3	5·2*	0·3	−0·1*	0·2	5·1	0·3	4·9*	0·3	−0·3*	0·1
HDL	1·12	0·11	1·16	0·12	0·04	0·03	1·25	0·10	1·30	0·10	0·05	0·05	1·02	0·41	1·01	0·05	−0·02	0·03
LDL	3·70	0·39	3·86	0·48	0·16	0·21	3·53	0·24	3·41	0·22	−0·12	0·17	3·50	0·32	3·19	0·28	−0·30	0·15
Plasma TAG (mmol/l)	1·66	0·41	1·77	0·41	0·11	0·15	1·07	0·20	1·05	0·14	−0·02	0·15	1·34	0·25	1·48	0·30	0·14	0·19

HOMA-IR, homeostasis model assessment for insulin resistance.

*Mean value was significantly different from that of the control group (*P* < 0·05).

†The effect of breakfast group was analysed using ANOVA for pre-intervention measures, and ANCOVA with the pre-intervention value as a covariate for the post-intervention and for the change (post–pre) in measures.

However, both post-intervention total cholesterol concentrations (*F*(2,32) = 4·895; *P* = 0·014) and change in total cholesterol concentrations (*F*(2,32) = 4·895; *P* = 0·014) were significantly different between the groups when controlling for pre-intervention total cholesterol concentrations. Pairwise comparisons revealed that post-intervention total cholesterol concentrations were significantly higher in the control group compared with both the cornflakes (*P* = 0·040) and oat porridge breakfast groups (*P* = 0·005), although the cornflakes and oat porridge groups did not differ from each other (Table 4). When pre-intervention values were controlled, there was no difference in post-intervention LDL- or HDL-cholesterol concentrations, or the change from pre- to post-intervention between any of the groups.

### Subjective ratings

#### Hunger ratings

**Fig. 1.**
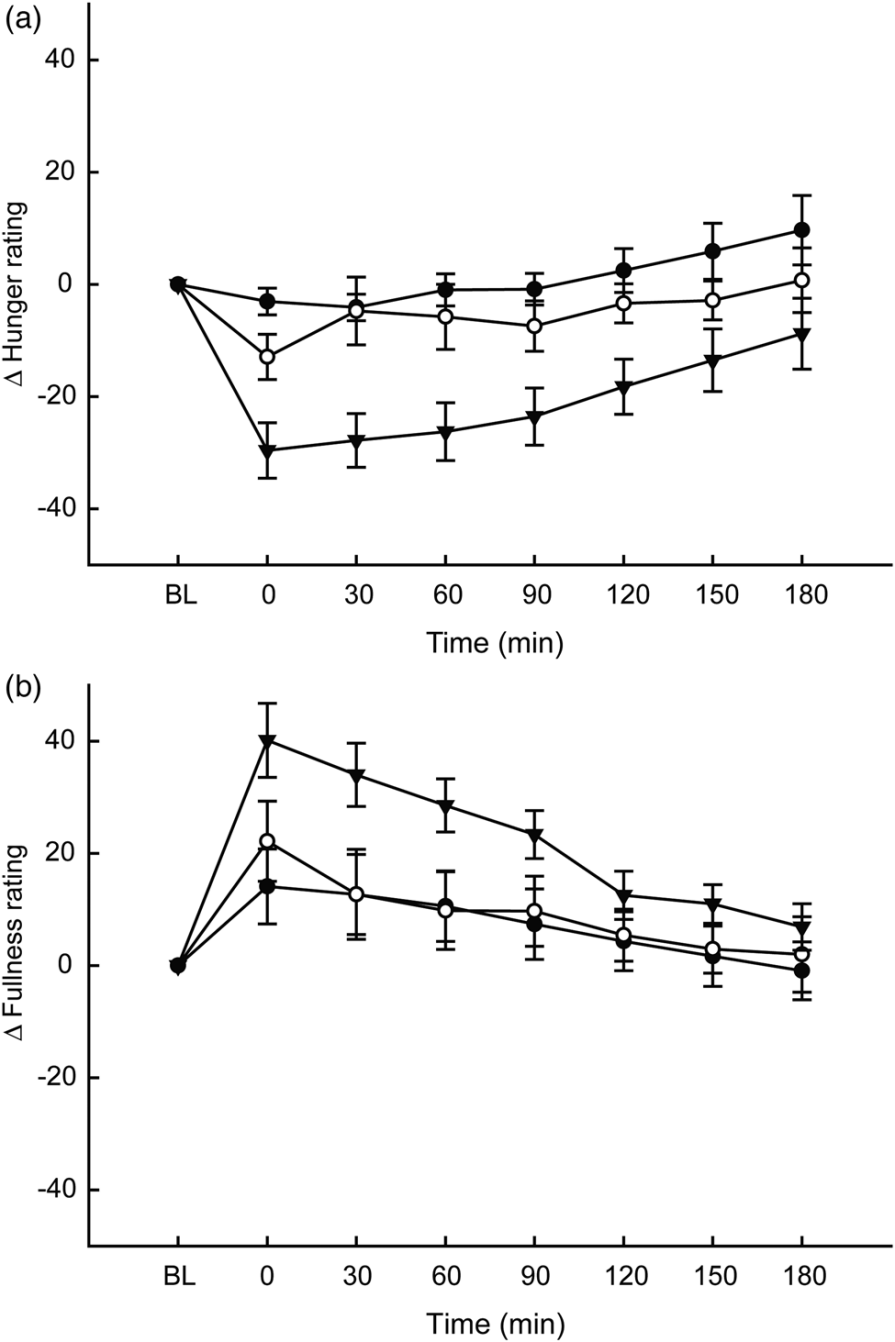
Changes compared with baseline (BL) in subjective hunger (a) and fullness (b) ratings over 4 weeks in response to breakfasts consisting of oat porridge (▾; *n* = 12), frosted cornflakes (○; *n* = 12) and water (control) (•; *n* = 12). Values are means, with standard errors represented by vertical bars. Between-within ANOVA showed that there were significant main effects of time (*P* < 0·001), breakfast group (*P* < 0·001) and time × breakfast group interaction (*P* = 0·003) hunger ratings. Fullness ratings displayed a significant main effect of time (*P* < 0·001) and breakfast × time interaction (*P* = 0·034) for fullness ratings.

Hunger ratings showed a significant main effect of time (*F*(7,33) = 11·31; *P* < 0·001), breakfast group (*F*(1,33) = 9·105; *P* < 0·001) and breakfast group × time interaction (*F*(14,33) = 2·43; *P* = 0·003), but there was no effect of week (main effect or interactions); thus mean ratings over the 4 weeks are displayed (Fig. 1(a)). Mean change in hunger ratings from the baseline were significantly lower in the oat porridge group compared with the control (*P* < 0·001) and cornflakes (*P* = 0·006) groups.

#### Fullness ratings

Fullness ratings showed a main effect of time (*F*(7,33) = 19·2; *P* < 0·001), and breakfast group × time interaction (*F*(14,33) = 1·84; *P* = 0·034), but there was no main effect of breakfast group or any effect of week (main effect or interaction); thus mean ratings over the 4 weeks are displayed (Fig. 1(b)). Mean change in fullness ratings from the baseline were significantly higher in the oat porridge group compared with in the control group (*P* = 0·036).

## Discussion

The primary aim of the present study was to compare the effect of consuming oat porridge, a high-fibre cereal, Frosted Flakes, a non-fibre cereal, and a no-breakfast (water) control for 4 weeks on changes in body weight and composition in overweight individuals. Additionally, we investigated the effects on subjective appetite ratings and CVD risk factors. The results showed that changes in body weight differed between the group that skipped breakfast (control), and both groups that consumed a fixed-energy breakfast (oat porridge or cornflakes), but there was no difference between the groups that consumed porridge or frosted cornflakes for breakfast. Body fat change did not differ between the groups, and there were also no changes in lean mass. This may be due to the limitation of air displacement as a measure of body composition, which can be influenced by total body water content^(^^26^^)^.

We propose that although skipping breakfast may result in an increase in later intake during the day, individuals may be unable to fully compensate for the energy from the missing meal, which results in a lower daily energy intake^(^^13^^)^. In turn, this lower overall daily energy intake could cause the weight loss seen in the control group that did not eat breakfast during the 4-week intervention. These differences in body weight were observed despite participants in the oat porridge group reporting lower hunger compared with the control and cornflakes groups, and higher fullness compared with the control group. It is possible that despite the differences in appetite ratings between the fixed-energy breakfast groups, there was no difference in energy intake later in the day between the oat porridge and cornflakes groups. This would be consistent with reports that although a high-fibre breakfast elicited greater fullness compared with a low-fibre breakfast, subsequent energy intake did not differ between the groups^(^^17^^,^^18^^)^.

It is important to note that although body-weight loss was greater in the control group relative to either the cornflakes or oat porridge groups, total cholesterol concentrations were also elevated in the control group that skipped breakfast. These findings are consistent with reports that skipping breakfast is associated with increases in cholesterol concentrations^(^^12^^,^^27^^)^. Furthermore, although there were no significant differences in change in total cholesterol concentrations between the cornflakes and oat porridge groups, the pattern for a greater reduction in cholesterol in the oat porridge group may be due, in part, to the presence of the β-glucan soluble fibre, which can reduce cholesterol concentrations^(^^20^^,^^28^^)^. There were no other between-group differences in the other CVD risk factors, which may be attributed in part to the relatively short study duration and small sample size.

Other limitations of the study include the absence of measures of total daily energy intake and physical activity patterns. Nevertheless, participants were requested not to change their usual activity routine during the study. Strengths of the present study include the careful supervision of subjects as they consumed the weekday breakfasts, and the careful matching of the two breakfast conditions for energy, weight, volume and energy density. However, despite matching for many attributes, the porridge breakfast contained 7 g more protein than the cornflakes breakfast, and although the total carbohydrate content of the porridge and frosted cornflakes was similar, the frosted cornflakes contained about 27 g more sugar. To match the volume of the two breakfasts, we incorporated water into the porridge and served water on the side of the frosted cornflakes breakfast. Even so, water served on the side may not be as filling as water incorporated in a food^(^^29^^)^.

In summary, the present study shows that in overweight individuals, skipping breakfast daily for 4 weeks leads to a reduction in body weight, but this is accompanied by an increase in total cholesterol concentrations compared with consuming either a frosted cornflakes or oat porridge breakfast. There were no differences in changes in body weight or total cholesterol concentrations between the groups consuming the frosted cornflakes no-fibre breakfast or the group that consumed the high-fibre oat porridge breakfast. These findings suggest that although skipping breakfast may be the more effective strategy to achieve weight loss than eating breakfast, there are associated detrimental effects on total cholesterol concentrations.

## Supplementary material

The supplementary material for this article can be found at http://dx.doi.org/10.1017/jns.2014.51
